# Secretome of Adipose Tissue as the Key to Understanding the Endocrine Function of Adipose Tissue

**DOI:** 10.3390/ijms23042309

**Published:** 2022-02-19

**Authors:** Damian Pogodziński, Lucyna Ostrowska, Joanna Smarkusz-Zarzecka, Beata Zyśk

**Affiliations:** Department of Dietetics and Clinical Nutrition, Medical University of Bialystok, Ul. Mieszka I 4B, 15-054 Bialystok, Poland; lucyna.ostrowska@umb.edu.pl (L.O.); joanna.smarkusz-zarzecka@umb.edu.pl (J.S.-Z.); beata.zysk@umb.edu.pl (B.Z.)

**Keywords:** proteomic, secretome, adipose tissue, fat tissue

## Abstract

The prevalence of obesity has reached pandemic levels and is becoming a serious health problem in developed and developing countries. Obesity is associated with an increased prevalence of comorbidities that include type II diabetes, cardiovascular diseases and some cancers. The recognition of adipose tissue as an endocrine organ capable of secreting adipokines that influence whole-body energy homeostasis was a breakthrough leading to a better molecular understanding of obesity. Of the adipokines known to be involved in the regulation of energy metabolism, very few are considered central regulators of insulin sensitivity, metabolism and energy homeostasis, and the discovery and characterization of new adipocyte-derived factors are still ongoing. Proteomics techniques, such as liquid chromatography-mass spectrometry or gas chromatography-mass spectrometry, have proven to be useful tools for analyzing the secretory function of adipose tissue (the secretome), providing insights into molecular events that influence body weight. Apart from the identification of novel proteins, the considerable advantage of this approach is the ability to detect post-translational modifications that cannot be predicted in genomic studies. In this review, we summarize recent efforts to identify novel bioactive secretory factors through proteomics.

## 1. Introduction

The prevalence of obesity has reached pandemic proportions with dramatic consequences for public health [1]. The increase in the number of overweight and obese individuals is the result of a sedentary lifestyle accompanied by a diet rich in simple carbohydrates and saturated fats which stimulates lipogenesis and leads to metabolic disorders in adipose tissue. An inactive lifestyle and an unhealthy diet constitute major risk factors for a variety of disorders, such as type 2 diabetes, non-alcoholic liver disease and cardiovascular disease [2,3]. Adipose tissue (AT), which is located beneath the skin and around larger organs, protects organs from mechanical damage. The majority of the adipose tissue mass is composed of adipocytes. Adipocytes contain a single lipid droplet in the cytoplasm and are characterized by a large lipid vacuole that fills most of the cell. The nucleus is pushed to the side, close to the cell membrane, and the cytoplasm forms a small ring around the vacuole [4]. Based on its location, fat tissue is divided into subcutaneous adipose tissue (SAT) and visceral adipose tissue (VAT), and other depots like mediastinic, perirenal, perigonadal, mesenteric, bone marrow and retroperitoneal, which differ in function and structure [4]. Subcutaneous adipocytes are large and less metabolically active, while visceral adipocytes are small and more active. Abdominal deposits, located beneath the abdominal visceral tissue, show the greatest lipolytic activity in both men and women. Catecholamine-induced lipolysis in VAT is primarily caused by an increase in the expression of β1 and β2 receptors, and an increase in the number and activity of β3 receptors, favoring the release of more fatty acids. Visceral adipocytes are also more sensitive to the action of hormones, such as estrogens, androgens, glucocorticoids and growth hormones [5,6]. A cluster of preadipocytes, fibroblasts, leukocytes, monocytes, macrophages, endothelial cells, and stem cell subpopulations constituting the stromal vascular fraction (SVF) help maintain the function of adipose tissue [7,8].

Adipose tissue functions as the body’s largest energy reservoir by storing energy in the form of triacylglycerols (TAGs). TAG release by adipose tissue is possible due to the action of hormone-sensitive lipase (HSL) and lipoprotein lipase (LPL). Through the action of HSL and LPL, TAGs are hydrolyzed to diacylglycerols (DAGs), which leads to the release of free fatty acids (FFA) into the blood plasma [9]. Of note, the activity of HSL is three times higher in adipose tissue than in the adrenal cortex [10]. Furthermore, HSL is more adaptable to lower temperatures—the enzyme is up to five times more active at 10 °C than LPL [11]. Thus, an increase in the amount of adipose tissue is associated with an increase in the concentration of FFA in the blood plasma. The activity of the enzyme is stimulated by insulin and increases with food consumption, in particular the intake of carbohydrate-rich foods.

There are several basic fatty acids: myristic, palmitic, stearic, oleic and linolenic acid. The relative proportions of these fatty acids in the body depend primarily on diet. Fatty acid uptake also depends, in part, on the presence of the very low density lipoprotein (VLDL) receptor. The receptor has an affinity for lipoproteins that are rich in apolipoprotein E, including chylomicrons and their residues and VLDL. By binding to TAG-rich lipoproteins, the VLDL receptor moves them in the proximity of LPL. Regardless of their origin, fatty acids need to be transported into cells and, despite the observation of FFA transport by a flip-flop mechanism in adipocytes, most of the released FFAs are transported by membrane proteins. These include CD36, fatty acid transport protein (FATP), fatty acid binding protein plasma membrane (FABPpm) and protocadherin fat (FAT) [12]. Although adipose tissue does not fit the conventional definition of an organ, it is considered an independent organ [4] due to its specific functions.

One of the most significant developments in obesity research was the recognition of adipose tissue as an endocrine organ. Adipocytes release many protein hormones and signaling factors, adipokines, which facilitate communication between adipose tissue and other organs, such as the liver and skeletal muscles [12]. It has been described that changes in these complex protein signatures play a key role in the pathophysiology of metabolic diseases [13]. Some of these proteins are inflammatory mediators thought to promote adipose tissue insulin resistance [14]. Others regulate insulin sensitivity directly. For example, adiponectin has been shown to increase insulin sensitivity in experimental models [15]. Of the body’s fat reservoirs, visceral fat is thought to be primarily responsible for metabolic disturbances in obesity [16]. This may be related to differences in adipokine secretion among adipose tissue depots [17]. Some adipokines are expressed predominantly by adipocytes. However, the SVF contributes to the endocrine and paracrine function of white adipose tissue (WAT) [18].

A transcriptome is a set of mRNA molecules (transcripts) present in a cell, group of cells or organism at a particular point in time. The transcriptome, unlike the genome, is dynamic. Cells respond to various factors by turning gene transcription on and off, thereby changing their transcripts. Transcriptome profiling in human adipose tissue has been used to successfully characterize obesity-related disorders, such as the report of enhanced expression of proinflammatory genes and proteins from the extracellular matrix (ECM) [19]. However, current data relating to the expression of potential adipokines by adipocytes and other adipose tissue cells is limited.

Proteins secreted by living cells into the extracellular space form the secretome, which is a rich and complex collection of molecules. Additionally, in some studies, the term “secretome” also includes molecules that are “shed” from the surface of living cells. Secreted proteins play a key role in cell signaling, communication and migration [20]. Intercellular communication, which is frequently mediated by secretory proteins, such as hormones, cytokines and chemokines, is critical for maintaining homeostasis in any multicellular organism. The efficient secretion and transmission of proteins influence basic processes such as metabolism and immunity. These processes involve the secretion of proteins in order to induce a paracrine response [21]. In many diseases, secreted proteins create conditions that lead to the development of further disorders, such as promoting cancer metastasis. Therefore, knowledge of the qualitative and quantitative composition of cellular secretomes is critical to understanding the biology of cellular interaction. This knowledge can be used to identify biomarkers for diseases.

The review aims to describe our current understanding of the human adipose tissue secretome, based on the results of proteomic studies, and attempts an explanation of the role of secreted proteins in adipose tissue in normal and pathological states.

## 2. Difficulties in Studying the Secretome

Proteins expressed by adipocytes cannot be studied in vivo, and therefore, in vitro culture is needed to study the secretome of adipocytes. Cultured adipocytes can be obtained by isolating them from mice, rats or humans or by differentiating the 3T3-L1 cell line.

Tissue explants, derived from living organisms, must be clear of lymph nodes [22,23], and then transported from the operating theater to the tissue culture laboratory. This creates additional logistical problems in terms of proper storage during transportation. Explants are normally stored in “transport buffer” (PBS, 5.5 mM glucose, 50 µg/mL gentamicin), which is a tissue transport medium that enables storage of collected tissue at room temperature [23]. Interestingly, it was difficult to find references describing explant transport methods in the available literature, which did suggest rapid isolation and seeding and the culturing of adipocytes onto scaffolds. 

Mouse 3T3-L1 pre-adipocyte cells are commercially available as a cell line. These are a useful research solution since the consent of an ethical committee is not required, as with explants.

Primary lines of human adipocytes or 3T3-L1 cells require an appropriate medium for the differentiation of cells into adipocytes. For cells isolated from human tissue, differentiation is induced by replacing DMEM/F12 medium with 10 mg/mL transferrin, for medium containing 2 nM T3, 100 nM insulin, 100 nM dexamethasone, 200 nM rosiglitazone and 540 mM 3-Isobutyl-1 -methylxanthine (IBMX) [24,25,26]. The 3T3-L1 cell lines should be differentiated according to the protocol provided by the manufacturer.

During cultivation, the adipocytes secrete the secretome into the culture medium, which itself contains proteins necessary for cell growth. Bovine or calf serum is also added to the medium to provide nourishment to cells or to induce differentiation. These proteins contained in the reagents contaminate the secretome, thus it is necessary to distinguish which proteins are the components of nutrients and sera, and which proteins have been secreted by adipose tissue [24,25].

One method is to replace a serum-enriched or differentiating medium with a medium devoid of glucose, insulin and serum [27]. This method does not allow for the examination of the proteins secreted during the earlier stages of the cell culture process. In addition, serum removal may alter the secretory activity and quality of the adipose tissue [28,29]. Another method that allows for distinguishing proteins contained in the culture medium from those secreted by cells is to specifically label newly synthesized proteins [21,22]. In order to perform an objective quantitative analysis of proteins secreted in serum-enriched media, stable isotope labeling by amino acids in cell culture (SILAC) [30,31] or azidohomoalanine (AHA) labeling can be utilized [32]. Cell labeling with a pulse of AHA, an azide-containing methionine analog, allows for selective and covalent capture of newly synthesized proteins in the medium. In pulsed SILAC (pSILAC), two parallel populations of cells are exposed to “intermediate” or “heavy” labeled arginine and lysine for a limited time. The relative quantities of various proteins that are produced during this time period are quantified by mass spectrometry. Moreover, the embedded SILAC label distinguishes newly produced proteins from preexisting ones [21]. 

## 3. Syndrome (SGBS) Cells

A growing body of evidence indicates that Simpson–Golabi–Behmel syndrome (SGBS) cells are an excellent tool for studying the secretome of adipocytes [33,34,35,36]. SGBS cells, which display morphological, physiological and biochemical similarity to human primary cells, are characterized by a higher capacity for differentiation, which they retain for at least 30 generations, compared to primary preadipocytes in culture [30,32]. Furthermore, it has been reported that SGBS cells differentiated in vitro behave in a similar manner to primary human adipocytes in the performance of functions, such as glucose transport, lipogenesis and lipolysis [34,36]. Based on genesis and gene expression analysis, differentiated SGBS cells are considered white subcutaneous adipocytes [35]. Therefore, SGBS cells are widely accepted and used in in vitro experiments with adipocytes. 

In a study by Qiao et al. [27], which was conducted using a peptide tagging method, 1326 proteins were identified, of which 230 were recognized by Deeploc as adipocyte-secreted proteins. Signal IP detected the presence of a signal peptide in 328 proteins. In total, the two groups represented 337 different proteins. Out of 337 proteins, 221 contained a signal peptide and were located in the extracellular space. 

In the same study, glucose restriction (GR) followed by refeeding (RF) of SGBS cells was performed as a simple in vitro surrogate for regaining weight in vivo after weight loss. The cells were cultured without glucose, and then the medium was supplemented with it. Changes in secreted proteins derived from human adipocytes were examined. Comparisons between normal feeding (NF) and GR plus RF were made to evaluate changes induced in the secretome that could serve as an indication of the consequences of weight regain for metabolic health. The study demonstrated that GR and RF induce adipocyte secretome changes involving biological pathways of ECM remodeling, lipid metabolism, the complement system and tissue homeostasis. Furthermore, 49 proteins secreted exclusively by adipocytes were described for the first time in the study [27].

The largest change in abundance due to the combined effect of GR and RF was an almost tenfold upregulation of ADAMTS-like protein 1 (ADAMTSL1), a metalloproteinase located in the ECM that is known for degrading aggrecan [37]. Aggrecan is a major structural macromolecule of cartilage responsible for the aggregation of hyaluronic acid. During aggrecan degradation by ADAMTSL1, changes in ECM consistency and poorer ion binding may occur, resulting in incorrect membrane polarity. Two other metalloproteinases: 72 kDa type IV collagenase (MMP2) and neutrophil collagenase (MMP8) were also upregulated, indicating that following GR and RF, the ECM is in a catabolic state. Furthermore, the three proteins involved in the maturation of collagen: prolyl 4-hydroxylase subunit alpha-1 (P4HA1), peptidyl-prolyl cis-trans isomerase C (PPIC) and serpin H1 (SERPINH1), were downregulated, resulting in slower development of fibers that maintain cell shape and anchor cellular organelles. Dysregulation of protein secretion indicates that adipose tissue may change to a looser structure, thus, enlarging its size. This could explain the expansion of adipose tissue after intensive weight loss—the looser the tissue, the larger the area. Another study showed that inside cells glucose restriction leads to the upregulation of some focal adhesion proteins [27]. This indicates that following GR and RF treatment, adipocytes enhance cell-cell interaction by going through a phase of increased ECM elasticity. Currently, it is not known whether enhanced ECM elasticity also occurs in organisms, i.e., in vivo, and whether it impacts weight regain or the metabolic state after weight regain. Nevertheless, in an earlier weight loss observation study, the expression of ADAMTSL1 and MMP2 genes was significantly increased four weeks after returning to a balanced diet [38], which confirms the hypothesis formulated through in vitro studies. This indicates that ECM adaptations also occur in vivo during weight loss and weight regain [27].

It is well known that adipose tissue secretes a number of components of the complement system. It is has been reported that changes in the secretion of complement factors during the development of overweight and obese states contribute to adipose tissue inflammation and the development of health complications [39]. Thus, it has been suggested that complement system modulation could be a target in the prevention and treatment of obesity-related metabolic diseases [40,41]. In an in vitro study by Qiao et al. [27], 12 proteins that influence complement activation were identified, eight of which were complement factors. Of these proteins, complement factor B (CFB) and complement factor 4B (C4-B) were significantly increased by GR and RF, while C1q and complement factor D (CFD) were significantly decreased. Such changes in vivo after weight loss and weight gain can lead to systemic changes in complement activity, particularly in individuals with increased adipose tissue mass [41].

In summary, in vitro observations indicate that changes in the complement system via GR and RF may trigger the uptake of triglycerides and glucose by adipocytes. In vivo gene expression studies have demonstrated that a poor ability to reduce myeloid adipose tissue activity after weight loss is associated with an increased risk of regaining weight [38,40].

A similar study was conducted by Renes et al. [42]. The study investigated the effect of reduced calorie intake (CR) on cells in vitro. Protein analysis revealed 34 proteins as classically secreted proteins, 22 as non-classical secreted proteins and 33 proteins as intracellular proteins. The category of classic secretory proteins was further broken down as ECM proteins (9), processing (4), regulatory/signaling proteins (18) and immune regulatory proteins (3). It was found that under the influence of CR, the number of most subunits and isoforms of collagen types I, III, IV, V and VI (COL1A1, COL1A3, COL1A4, COL1A5, COL1A6), and fibronectin (FN) decreased. This was probably due to the overall reduction in cell size caused by a decline in the number of TAGs [43,44]. One of the main functions of adipose tissue is the storage of TAGs. Increased uptake of fat by mature adipocytes leads to their growth [45]. When calorie intake is limited, and thus fat uptake is reduced, large volume adipocytes stop storing fats, which leads to a decrease in their size and results in cell reconstruction [45]. Overall, reducing the size of adipocytes results in decreased expression of structural proteins.

The study also revealed that CR caused upregulation of the structural proteins cofilin 1, transgelin and vimentin. One of the basic functions of these proteins is the reorganization of the cell’s cytoskeleton [42]. Such a relationship should not be surprising—reducing the size of cells leads to the reorganization of their structure, and thus to greater expression of structural proteins.

A decrease in TAGs was also observed in a study by Rosenow et al. [46] in which SGBS cells were treated with resveratrol (RSV). RSV is a natural compound found in some plants, such as red grapes, which possesses a high antioxidant potential and functions in the modulation of lipid metabolism [47,48]. A decrease in the number of structural proteins, such as vimentin or SERPINH1 was also shown, while proteins processing the cell structure, such as cathepsin L1 or cystatin-C1, were upregulated. Thanks to this, it is possible to remodel the cell after reducing its size by reducing the TAG content [46]. 

## 4. Exosomes-Secretory Vesicles in Adipose Tissue

Exosomes are predominantly multi-vesicular body (MVB) vesicles that are 40–150 nm in size, which contain microRNAs and numerous molecules involved in signal transduction, such as protein kinases and heterotrimeric G proteins, various metabolic enzymes and the heat shock proteins Hsp70 and Hsp90 [49]. Microbubbles, which are typically between 50 nm and 1000 nm in diameter, come from the cell membrane and are involved in the secretion of proteins [50]. Within the field, unconventional protein secretion (UPS) has been associated exclusively with cellular stress, e.g., of apoptotic bodies; however, growing evidence suggests that it is a form of regular intercellular communication [51].

Proteomic profiling of exosomes derived from primary human adipocytes identified 884 exoadipokines [3]. Comparison of the exoadipokine list with the entire human subset of ExoCarta showed that 92% of the proteins had already been described in the context of exosomal secretion. ExoCarta is a compendium containing datasets from various exosome studies. Unfortunately, a lack of data specific to human adipocytes or adipose tissue limits tissue-specific comparisons. Nevertheless, 67 novel exoadipokines were identified. The identified proteins are well-known adipokines such as adiponectin, fatty acid binding protein 4 (FABP4) and several metalloproteases. Since most of the exosomal proteins released from adipocytes are listed in ExoCarta, it can be assumed that most of the proteins released from adipose tissue are also released by adipose tissues in vivo. This could mean that all organs communicate with each other and that only a small number of released proteins is unique to each tissue, with the majority of proteins being common to most tissues [52].

One example of an adipokine released by exosomes via a non-classical pathway is FABP4. It is found in the exosomes of primary adipocytes. A number of studies in murine models have demonstrated that the FABP4 protein is commonly found in adipocytes and may contribute to obesity-related metabolic diseases, such as atherosclerosis, type 2 diabetes and non-alcoholic fatty liver disease [52,53]. FABP4 is an adipose tissue-specific protein and may serve as a specific marker of adipocyte-derived exosomes. FABP has been linked to intracellular metabolic activity, although some evidence suggests that the protein, which is released exosomally, has considerable significance as a circulating adipokine [54,55]. Analysis of exosome content, including protein, lipid, or microRNA content, may help define the function of adipose tissue without the need for adipose tissue biopsy, and it may enhance the understanding of signals reaching other organs from enlarged fat tissue during inflammation [3].

Many exoadipokines found in the pathways specifically associated with liver fibrosis, liver lesions, or liver cancer are components of the ECM or regulate the synthesis and structure of the ECM. Adipose tissue in obesity is characterized not only by inflammation but also by fibrosis, which is associated with the development of obesity-related metabolic diseases [56,57]. Therefore, it may be postulated that some exoadipokines are associated with fibrosis directly in adipose tissue and the liver. In fact, several studies have demonstrated that exosomes of various origins, including adipose tissue, are taken up by the liver [58,59,60]. In vitro evidence indicates that adipose tissue exosomes can directly regulate the mediators of the TGF β pathway in liver cells, which is associated with liver fibrosis [61]. Interestingly, Ingenuity™ pathway analysis showed that major pathways involved in liver fibrosis, such as integrin signaling [62], are related to exosome content [3]. In addition, proteins related to the mTOR pathway, which is a well-known regulator of metabolism and also a target for liver fibrosis, are enriched in adipocyte-derived exosomes.

## 5. Other Depots of Adipose Tissue—Gonadal Adipose Tissue and Perivascular Adipose Tissue

One of the depots of adipose tissue is gonadal fat (GAT), which surrounds the gonads [63,64]. In a study by Roca-Rivada et al. [63], visceral, subcutaneous and gonadal adipose tissue was examined proteomically, respectively. The authors identified 188, 85 and 91 proteins, respectively, [63] in the tissues. Known adipose tissue adipokines found included adiponectin (ADIPOQ), retinol binding protein 4 (RBP4), angiotensinogen (AGT), macrophage migration inhibitory factor (MIF), haptoglobin (HP) and serum amyloid P component (APCS). In terms of the secretome, gonadal and visceral fat were similar, while subcutaneous fat differed from the first two [63]. The proteins produced by gonadal adipose tissue included fumarin hydratase, 3-ketoacyl-CoA thiolase (ACAA2), alpha-enolase (ENO1), gelsolin (Gsn), serotransferrin (Tf) and cathepsin D (Ctsd) [63].

Similar results were obtained in a study by Pardo et al. [64]. Based on differential analysis, proteins, such as enoyl-CoA hydratase (Echs1), adenosine kinase (Adk) and transgelin were identified only in the secretions of visceral and gonadal adipose tissue [64]. This confirms that gonadal and visceral fat are similar in terms of secretion and proteomic profiles.

Another depot of adipose tissue is perivascular adipose tissue (PVAT). PVAT surrounds most blood vessels except capillaries and pulmonary and cerebral blood vessels [65]. PVAT plays a wide range of physiological roles that go beyond supporting connective tissue. Currently, it is considered a metabolically active organ that regulates both autocrine and paracrine vascular function by producing various adipokines [66].

Paracrine penetration between PVAT and adjacent arteries, also known as “vasocrine” communication, actively regulates vasculitis and arterial remodeling [67]. Most PVAT-generated cytokines, such as tumor necrosis factor (TNF-α), interleukin-6 (IL-6), plasminogen activator inhibitor-1 (PAI-1) and monocyte chemoattractant protein-1 (MCP-1), are proinflammatory and atherosclerotic [65]. Adiponectin (ADIPOQ) is one of the few anti-inflammatory agents that play a role in preventing cardiovascular disease [68].

Many of the factors derived from PVAT are also vascular tension regulators. Such a factor was first discovered in 2002, adipocyte-derived relaxation factor (ADRF) [69]. Other relaxation factors derived from PVAT include adiponectin, gaseous molecules (e.g., NO and H2S), methyl palmitate, AGT and omentin/intellectin (ITLN) [65]. Depending on the location of PVAT and other various conditions, some agents, including H2S [70], leptin (LEP), TNF-α, IL-6 and apelin, may act as vasodilators or constricting agents [65,71]. By way of illustration, leptin can induce vasodilation to modulate blood pressure homeostasis [72], while obesity-induced hyperleptinemia causes an increase in endothelin-1, which then leads to vasoconstriction [73]. Some reports have shown that TNF-α causes vasodilation through the production of nitric oxide [71]. On the other hand, TNF-α can also constrict blood vessels by increasing endothelin-1 (EDN1) levels [74].

## 6. The Secretome of Brown Adipose Tissue—Batokines

In recent years, considerable research has clarified the role of brown adipose tissue (BAT) as a secretory organ, including its endocrine function. BAT, similar to WAT, is believed to play a role in whole-body energy expenditure as it is the major contributor to non-shivering thermogenesis. In brown adipocytes, there is a regulated uncoupling of the mitochondrial respiratory chain from ATP synthesis resulting in thermogenesis; the unique presence of uncoupling protein-1 (UCP1) in brown adipocyte mitochondria is responsible for this phenomenon and specializes brown adipocytes to converts the energy of substrate oxidation into heat instead of ATP [75]. A comprehensive understanding of the physiological mechanisms regulating BAT as a system for generating heat in response to environmental stimuli has emerged [76]. Of note, there is some inaccuracy in the use of the term “brown adipokines”, also called “batokines”, which results from the use of the general term “adipokine”. For years, none of the molecules reported to be released by BAT were specific, i.e., they were not released solely by BAT. Hence, in the literature, brown adipokines are often classified as factors that are “preferentially” secreted by BAT compared to WAT [75]. 

Considering the advances in proteomics, it seems that direct assessment of the proteins released by brown adipocytes would be a preferred analytical approach for gaining sound knowledge of the brown adipocyte secretome. However, only two studies using this strategy have been published to date [77,78]. The studies identified a set of proteins whose expression was significantly induced in a mouse BAT culture. Findings from these studies are remarkably consistent, with 60% (42 out of 71) of the proteins found in a study by Villarroya et al. [77] also identified in a study by Ali Khan et al. [22]. Some of these factors are adipokines that are already known to be secreted by brown adipocytes, such as ADIPOQ and AGT [78,79]. Other, previously identified brown adipokines, such as fibroblast growth factor-21 (FGF21), neuregulin-4 (NRG4), or bone morphogenetic protein-8b (BMP8b) [80] were not found in either of the two studies. However, it is noteworthy that only five proteins are commonly recognized as batokines: two components of the ECM (collagen alpha-1(III) chain-COL3A1 and procollagen C-endopeptidase enhancer 1-PCOLCE), insulin-like growth factor 4 binding protein (IGFBP4), follistatin-like protein 1 (FSTL1) and chemerin [75]. 

**Figure 1:**
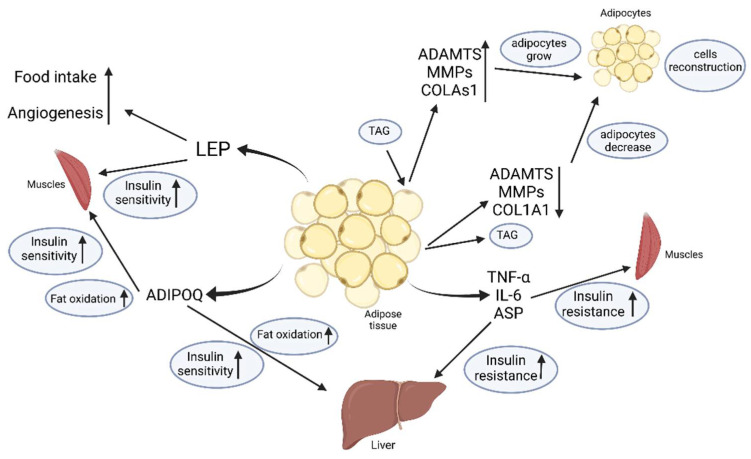
Selected adipokines and their effects on individual organs. LEP-leptin; ADIPOQ-adiponectin; TNF-α- Tumor necrosis factor α; IL-6-interleukin 6; ADAMTS- disintegrin and metalloproteinase with thrombospondin motifs; MMPs-matrix metalloproteinases; COL1A1-collagen alpha-1(I) chain; ASP-Acylation stimulating protein.

Figure 1 and Table 1 summarize the main components of the adipose tissue secretome and related mechanism.

## 7. Summary

Despite the considerable genetic and proteomic knowledge of adipose tissue, there are proteins produced by this tissue that have not yet been identified. Difficulties relating to the study of secretory proteins—the secretome—contribute to the challenges associated with determining their roles and defining the quantities released in normal and pathological states. Therefore, in vitro cultures are utilized to examine proteins secreted by cells. However, they remain a model rather than a fully functional tissue, and difficulties in collecting the secretome make it impossible to thoroughly study the metabolic pathways of adipose tissue.

## Figures and Tables

**Table 1:** Selected secretory protein with function and tissue/cells where it was identified.

Protein Name	Role from UniProt KB	Secret Tissue/Cells	Citation
3-ketoacyl-CoA thiolase, mitochondrial (ACAA2)	this is one of the enzymes that catalyzes the last step of the mitochondrial beta-oxidation pathway, an aerobic process breaking down fatty acids into acetyl-CoA (probable)	GAT	Roca-Rivada et al. [63]
72 kDa type IV collagenase (MMP2)	remodeling of the vasculature, angiogenesis, tissue repair, tumor invasion, inflammation	SAT; SGBS	Hartwig et al. [3]; Qiao et al. [27]
ADAMTS-like protein 1 (ADAMTSL1)	metalloendopeptidase activity; extracellular matrix organization	SAT; SGBS	Hartwig et al. [3]; Qiao et al. [27]
Adenosine kinase (Adk)	ATP dependent phosphorylation of adenosine and other related nucleoside analogs to monophosphate derivatives	VAT; GAT	Roca-Rivada et al. [63]; Pardo et al. [64]
Adiponectin (ADIPOQ)	adipokine involved in the control of fat metabolism and insulin sensitivity, with direct anti-diabetic, anti-atherogenic and anti-inflammatory activities	VAT; SAT; BAT; SGBS; GAT; PVAT	Alvarez-Llamas et al. [23]; Hartwig et al. [3]; Khan et al. [22]; Roca-Rivada et al. [63]; Pardo et al. [64]; Renes et al. [42]; Rosenow et al. [46] et al.; Li et al. [65]
Alpha-enolase (ENO1)	catalyzes the conversion of 2-phosphoglycerate to phosphoenolpyruvate	GAT	Roca-Rivada et al. [63]
Angiotensinogen (AGT)	essential component of the renin-angiotensin system (RAS), a potent regulator of blood pressure, body fluid and electrolyte homeostasis	VAT; GAT	Roca-Rivada et al. [63]
Bone morphogenetic protein 8B (BMP8b)	induces cartilage and bone formation	BAT	Villarroya et al. [76]
Cathepsin D (Ctsd)	active in intracellular protein breakdown	VAT; GAT; SGBS	Alvarez-Llamas et al. [23]; Qiao et al. [27]; Roca-Rivada et al. [63]; Pardo et al. [64]
Cathepsin L1 (CTSL1)	plays a critical for normal cellular functions such as general protein turnover, antigen processing and bone remodeling	VAT; SGBS	Alvarez-Llamas et al. [23]; Qiao et al. [27]; Renes et al. [42]; Rosenow et al. [46]
Collagen alpha-1(I) chain (COL1A1)	it is a member of group I collagen (fibrillar forming collagen)	VAT; SAT; SBGS; BAT	Alvarez-Llamas et al. [23] Renes et al. [42] Rosenow et al. [46] Khan et al. [22]
Collagen alpha-1(III) chain (COL3A1)	involved in regulation of cortical development	VAT; SAT; SGBS; BAT	Alvarez-Llamas et al. [23]; Dahlman et al. [18]; Khan et al. [22]; Renes et al. [42]
Collagen alpha-1(V) chain (COL5A1)	it is a minor connective tissue component of nearly ubiquitous distribution	SAT; SGBS	Dahlman et al. [18]; Renes et al. [42]
Complement C4-B (C-4B)	non-enzymatic component of the C3 and C5 convertases and thus essential for the propagation of the classical complement pathway	SAT; SGBS	Hartwig et al. [3]; Qiao et al. [27]
Complement factor B (CFB)	it is part of an alternative complement pathway	SAT; SGBS	Dahlman et al. [18]; Hartwig et al. [3]; Qiao et al. [27]
Complement factor D (CFD)	selective cleavage of Arg-|-Lys bond in complement factor B	SAT; SGBS	Dahlman et al. [18]; Hartwig et al. [3]; Qiao et al. [27]
Cystatin-C1 (CST3)	inhibitor of cysteine proteinases	VAT; SAT	Alvarez-Llamas et al. [23]; Dahlman et al. [18]; Rosenow et al. [46]
Enoyl-CoA hydratase, mitochondrial (Echs1)	straight-chain enoyl-CoA thioesters from C4 up to at least C16 are processed, although with decreasing catalytic rate	VAT; GAT	Roca-Rivada et al. [63]; Pardo et al. [64]
Fatty acid-binding protein (FABP4)	lipid transport protein in adipocytes.	SAT; BAT; VAT	Hartwig et al. [3]; Khan et al. [22]; Roca-Rivada et al. [63]
Fibroblast growth factor 21 (FGF21)	stimulates glucose uptake in differentiated adipocytes via the induction of glucose transporter SLC2A1/GLUT1 expression	BAT	Villarroya et al. [76]
Fumarate hydratase, mitochondrial (FH)	catalyzes the reversible stereospecific interconversion of fumarate to L-malate	GAT	Roca-Rivada et al. [63]
Galectin-1 (LGALS1)	binds beta-galactoside and a wide array of complex carbohydrates	SGBS; VAT; GAT; SAT	Qiao et al. [27]; Roca-Rivada et al. [63]; Pardo et al. [64]
Gelsolin (Gsn)	actin-modulating protein that binds to the plus (or barbed) ends of actin monomers or filaments	VAT; GAT; SGBS	Alvarez-Llamas et al. [23]; Roca-Rivada et al. [63]; Qiao et al. [27]
Haptoglobin (HP)	captures, and combines with free plasma hemoglobin to allow hepatic recycling of heme iron and to prevent kidney damage	VAT, GAT	Roca-Rivada et al. [63]
Leptin (LEP)	regulator of energy balance and body weight control; once released into the circulation, has central and peripheral effects	VAT; BAT; GAT; PVAT	Alvarez-Llamas et al. [23]; Villarroya et al. [76]; Roca-Rivada et al. [63]; Li et al. [65]
Macrophage migration inhibitory factor (MIF)	Involved in the innate immune response to bacterial pathogens	VAT; GAT	Roca-Rivada et al. [63]
Neutrophil collagenase (MMP8)	degradation fibrillar type I, II, and III collagens	SGBS	Qiao et al. [27]
Nicotinamide phosphoribosyltransferase (NAMPT)	Catalyzes the condensation of nicotinamide with 5-phosphoribosyl-1-pyrophosphate to yield nicotinamide mononucleotide, an intermediate in the biosynthesis of NAD	PVAT	Li et al. [65]
Peptidyl-prolyl cis-trans isomerase C (PPIC)	catalyzes the cis-trans isomerization of proline imidic peptide bonds in oligopeptides	SGBS	Qiao et al. [27]
Prolyl 4-hydroxylase subunit alpha-1 (P4HA1)	Post-translational catalysis of 4-hydroxyproline formation in collagens	SGBS	Qiao et al. [27]
Pro-neuregulin-4 (NRG4)	ligand for the ERBB4 tyrosine kinase receptor	BAT	Villarroya et al. [76]
Retinol-binding protein 4 (RBP4)	retinol-binding protein that mediates retinol transport in blood plasma	SAT; SGBS; GAT	Hartwig et al. [3]; Qiao et al. [27]; Roca-Rivada et al. [63]
Serotransferrin (Tf)	iron binding transport proteins which can bind two Fe^3+^ ions in association with the binding of an anion	VAT; GAT	Alvarez-Llamas et al. [23]; Pardo et al. [64]
Serpin H1 (SERPINH1)	binds specifically to collagen	SGBS	Qiao et al. [27]
Serum amyloid P-component (APCS)	can interact with DNA and histones and may scavenge nuclear material released from damaged circulating cells	VAT; GAT	Roca-Rivada et al. [63]
Transgelin (Tagln)	Actin cross-linking/gelling protein	VAT; GAT; SGBS	Roca-Rivada et al. [63]; Pardo et al. [64]; Renes et al. [42]
Tumor necrosis factor (TNF)	can induce cell death of certain tumor cell lines	VAT; SGBS; PVAT	Alvarez-Llamas et al. [23]; Rosenow et al. [46] et al.; Li et al. [65]
Vimentin (VIM)	class-III intermediate filaments found in various non-epithelial cells	SGBS	Roca-Rivada et al. [63]; Renes et al. [42]; Rosenow et al. [46] et al.

## Data Availability

Not applicable.
